# A stage IV lung squamous cell cancer patient with brain metastases, high PD-L1 & TMB, achieves pCR and long-term survival after immune-chemotherapy and radical surgery: a case report and literature review

**DOI:** 10.3389/fimmu.2025.1601125

**Published:** 2025-07-04

**Authors:** Xiangyu Xu, Zheng Ma

**Affiliations:** Department of Thoracic Surgery, Chongqing General Hospital, Chongqing University, Chongqing, China

**Keywords:** non-small cell lung cancer (NSCLC), brain metastasis, programmed cell death ligand 1, tumor mutational burden (TMB), pathological complete response (PCR), circulating tumor DNA (ctDNA)

## Abstract

Brain metastases occur in 40% of advanced NSCLC patients, with poorer prognosis in squamous subtypes. Immune checkpoint inhibitors (ICIs) combined with chemotherapy have revolutionized treatment, yet data on the systematic treatment of stage IV squamous non-small cell lung cancer with surgery remain limited. A 59-year-old male smoker presented with stage cT4N2M1b IVA squamous NSCLC and a solitary brain metastasis. Next-generation sequencing revealed programmed cell death ligand 1 (PD-L1) high expression (TPS=75%) and TMB-High (28.49 Mut/Mb) without driver mutations. After pembrolizumab plus platinum-based chemotherapy induced conversion therapy for 3 cycles, the brain lesion achieved pathological complete response (pCR) following resection, while the primary lung tumor showed major pathological response (MPR) post-surgery. Postoperative adjuvant chemoimmunotherapy and 2-year pembrolizumab maintenance were administered. Serial circulating tumor DNA (ctDNA) monitoring remained negative, with no recurrence observed over 50 months. This is the first reported case of long-term survival (PFS >50 months) in a PD-L1-high/TMB-High squamous NSCLC patient with brain metastasis treated with immunotherapy-based multimodal therapy. Our findings suggest that biomarker-guided strategies integrating systemic therapy, surgery, and MRD monitoring may enable curative potential in select advanced NSCLC patients. Further studies are warranted to validate this “sandwich” approach (drug-surgery-drug) and optimize treatment duration.

## Introduction

Advanced non-small cell lung cancer (NSCLC) frequently metastasizes to distant sites, with brain involvement observed in 40% of cases (1). Treatment-naïve patients with driver gene-positive tumors exhibit a higher incidence of brain metastases (30%-40%) compared to driver gene-negative counterparts (15%-35%), during systemic therapy, the cumulative incidence of brain metastases escalates to 60% (2). Squamous cell carcinoma (SqCC) demonstrates a lower propensity for intracranial spread (7%) relative to adenocarcinoma (3). Local interventions, including surgery and stereotactic radiosurgery (SRS), remain cornerstone strategies for NSCLC brain metastases, conferring survival benefits (4). However, the recurrence rate of brain metastases within one year post-surgery is as high as 50–60% (5), and combination therapies may reduce intracranial recurrence.

The prognosis of NSCLC with brain metastases is generally poor, with a median OS of approximately 17 months for adenocarcinoma patients (6) and 8 months for other NSCLC subtypes (7). However, with the rapid development and remarkable efficacy of targeted therapy and immunotherapy (8, 9), some patients with unresectable advanced NSCLC can be converted to an operable state after systematic treatment, providing an opportunity for long-term survival for patients with lung cancer brain metastases. Herein, we present a case of brain metastatic pulmonary SqCC with high programmed cell death ligand 1 (PD-L1, tumor proportion score [TPS]=75%, combined positive score [CPS]=75) expression and high tumor mutational burden (TMB-H, 28.49 Mut/Mb) who underwent sequential resection of brain and lung lesions after immunotherapy combined with chemotherapy. The patient achieved pathological complete response (pCR) in the brain metastasis and major pathological response (MPR) in the primary lung tumor. Postoperative longitudinal molecular residual disease (MRD) monitoring and follow-up have shown no recurrence, with progression-free survival (PFS) exceeding 50 months.

## Case description

**Figure 1:**
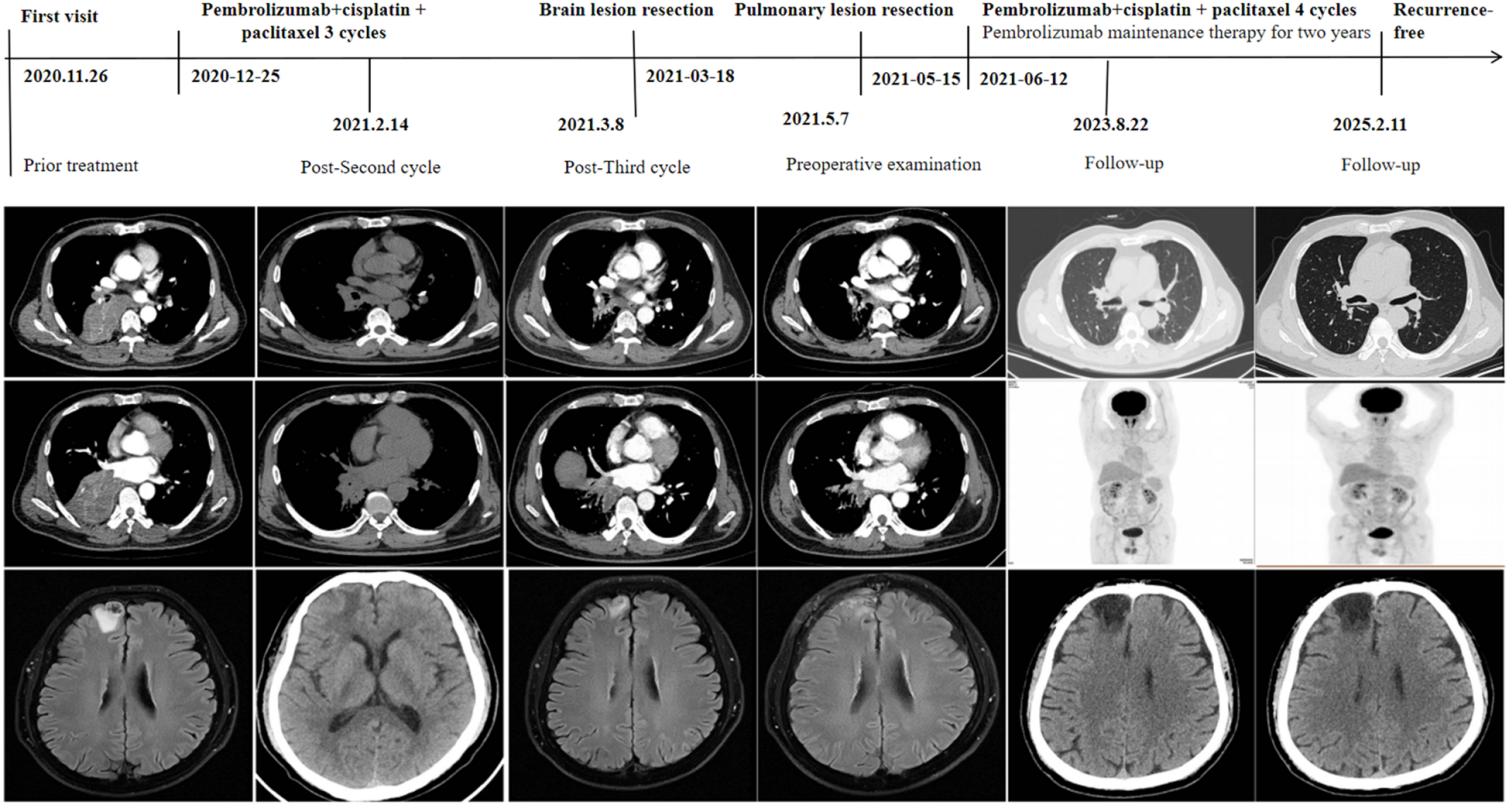
Treatment flow and imaging findings.

**Figure 2:**
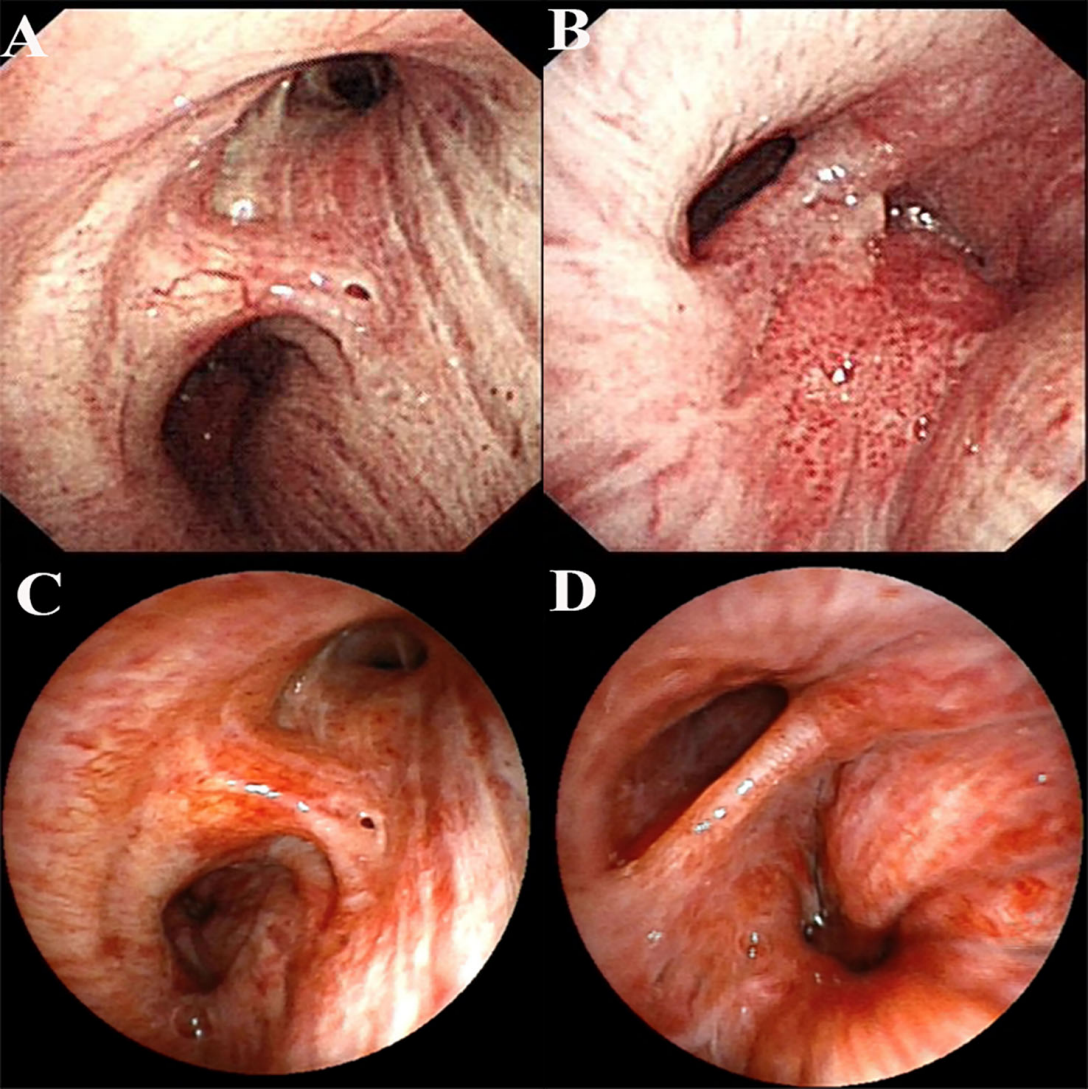
Bronchoscopic images before and after treatment. **(A)** Bronchoscopic image before treatment (right subcarina); **(B)** Bronchoscopic image before treatment (right intermediate bronchus); **(C)** Bronchoscopic image after conversion therapy (right subcarina); **(D)** Bronchoscopic image after conversion therapy (right intermediate bronchus).

**Figure 3:**
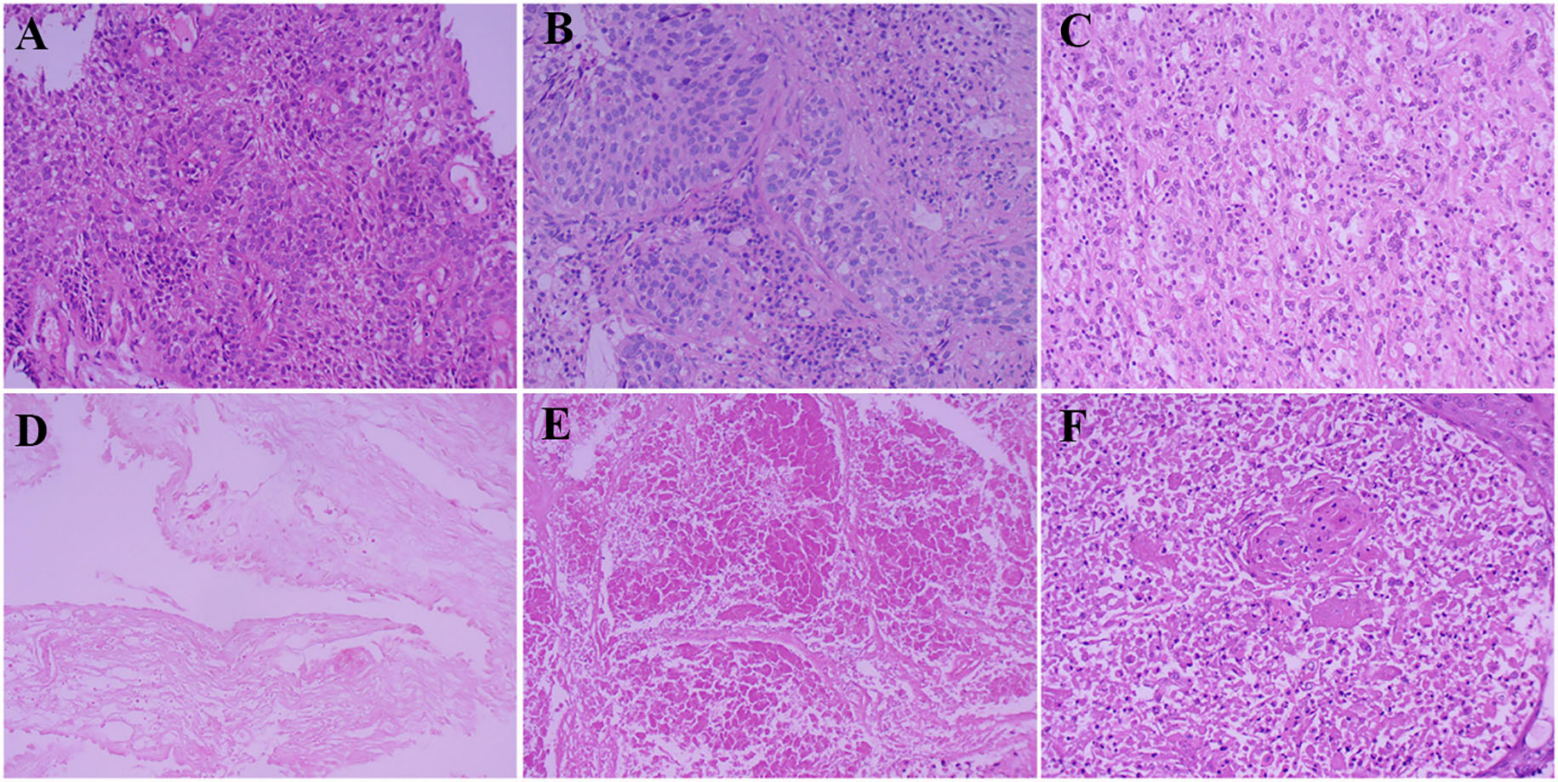
Pathological results of specimens before and after surgery. **(A)** Bronchoscopic specimen before conversion therapy; **(B)** Percutaneous biopsy specimen before conversion therapy; **(C)** Resected specimen of frontal lobe lesion after treatment; **(D)** Bronchoscopic specimen after conversion therapy; **(E)** Lung tissue specimen after surgery; **(F)** Specimen of group 11 lymph nodes after surgery.

A 59-year-old male presented to our hospital on November 26, 2020, following the detection of a right lower lung mass during a routine health checkup one week prior. The patient had no history of chronic diseases but reported a 40-year smoking history (20 cigarettes/day) and occasional alcohol consumption. Physical examination was unremarkable. Breath sounds were normal without obvious abnormalities, and no palpable enlargement of superficial lymph nodes was noted. Admission chest contrast-enhanced computed tomography (CT) demonstrated occlusion of the right lower lobar bronchus, collapse of the right lower lobe with a mass-like lesion (8.9 cm × 7.8 cm), invasion and occlusion of the right inferior pulmonary vein, narrowing of partial branches of the right inferior pulmonary artery, and mild stenosis of the right middle lobar bronchus (**Figure 1**). Enlarged mediastinal and right hilar lymph nodes were observed, most notably a subcarinal lymph node measuring 2.9 cm × 2.1 cm (**Figure 1**). These findings suggested a neoplastic lesion (suspected lung cancer) in the right lower lobe accompanied by lobar collapse, mediastinal/hilar lymph node metastases, and vascular involvement. Fiberoptic bronchoscopy revealed stenosis at the right middle lobar orifice and a neoplasm at the right lower lobar orifice, located 3 cm distal to the carina and 1.5 cm from the right upper lobar orifice (**Figures 2A, B**). Endoscopic biopsy indicated severe squamous dysplasia with papillary growth (**Figure 3A**). Percutaneous lung biopsy confirmed squamous cell carcinoma (**Figure 3B**). Brain contrast-enhanced magnetic resonance imaging (MRI) identified a right frontal lobe lesion (1.6 cm × 1.2 cm) (**Figure 1**), consistent with lung cancer metastasis. Whole-body bone scintigraphy and abdominal ultrasonography showed no distant metastases. The patient was staged as cT4N2M1b IVA. Next-generation sequencing (NGS) and immunohistochemistry revealed no driver gene mutations, high PD-L1 expression (TPS=75%, CPS=75; DAKO 22C3 antibody) and high TMB (28.49 mutations/megabase [Mut/Mb]) from puncture tissue sample.

Following multidisciplinary team (MDT) discussion (thoracic surgery, oncology, pulmonology, radiotherapy) and informed consent, the patient initiated anti-PD-1 therapy combined with platinum-doublet chemotherapy (pembrolizumab 200 mg IV, cisplatin 75 mg/m² + paclitaxel 135 mg/m² on day 1 every 3 weeks) on December 25, 2020. After one treatment cycle, chest X-ray (digital radiography, DR) showed reduced opacity in the right lower lung field. Post-cycle 2 imaging (February 14, 2021) revealed shrinkage of the right lower lobe lesion (4.9 cm × 3.5 cm) (**Figure 1**), alleviated bronchial obstruction, and decreased subcarinal lymph node short axis to 1.81 cm (**Figure 1**). Brain CT demonstrated a nodular isodense lesion in the right frontal lobe (**Figure 1**). After three cycles (March 8, 2021), chest contrast-enhanced CT showed further reduction of the pulmonary lesion (3.6 cm × 3.3 cm) (**Figure 1**) and subcarinal lymph node (short axis: 1.7 cm), with persistent vascular involvement. Brain MRI revealed a smaller right frontal lesion (0.8 cm × 0.6 cm) (**Figure 1**) with reduced density. Positron emission tomography-computed tomography (PET-CT) demonstrated a soft tissue mass (3.58 cm × 3.29 cm, SUVmax 4.09) in the right lower lobe hilum, enlarged subcarinal lymph nodes (short axis 1.42 cm, SUVmax 4.7), and a right frontal hypodense lesion (1.45 cm, SUVmax 7.41) without other metastases. Response Evaluation Criteria in Solid Tumors (RECIST) version 1.1 indicated partial response (PR). The patient developed grade 1 myelosuppression during treatment, managed with granulocyte colony-stimulating factor, without grade 3–5 adverse events. Repeat MDT assessment confirmed resectability of the right lower lung lesion. Five weeks after cycle 3 (March 18, 2021), the patient underwent navigated right frontal metastasectomy. Postoperative pathology revealed chronic inflammation, reactive gliosis, and keratin-positive cellular debris, consistent with treated metastatic carcinoma (**Figure 3C**), according to the Expert Consensus of Chinese Medical Association achieved pCR, which have no residual viable tumor cells following conversion therapy (10). Re-admitted on May 6, 2021, preoperative chest/abdominal CT (May 7, 2021) showed a residual right lower lobe lesion (3.1 cm × 3.9 cm) with stable lymphadenopathy (**Figure 1**). Brain MRI and bone scintigraphy revealed no metastases (**Figure 1**). Repeat bronchoscopy demonstrated mucosal protrusion and stenosis at the right lower lobar orifice, with biopsy showing chronic inflammation (**Figures 2C, D**). Restaged as ycT2aN2aM0 IIIA, the patient underwent thoracoscopic right middle-lower lobectomy with mediastinal lymphadenectomy and partial pericardiectomy on May 15, 2021, due to intraoperative findings of middle lobe invasion.

**Figure 4:**
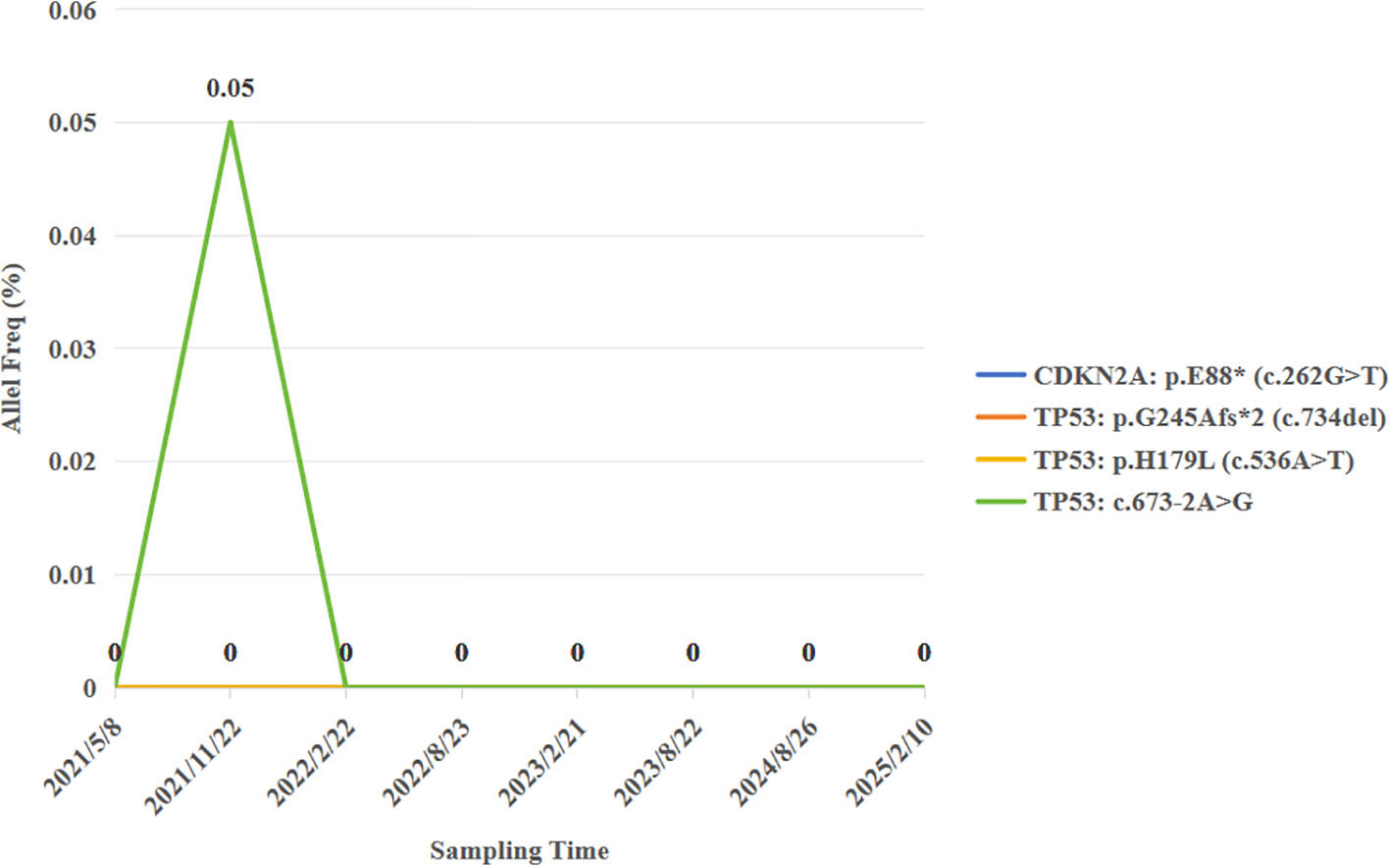
Postoperative MRD monitoring.

Pathological examination identified a 2.5 cm × 2.2 cm tumor bed with no residual carcinoma (**Figures 3D, E**), absent lymphovascular or perineural invasion, and bronchial stump without cancer. Lymph nodes submitted for pathological examination included groups 2R (0/2), 4R (0/11), 7 (0/3), 10 (0/1), 11 (1/3), and 12 (0/1). Residual tumor was detected at 1 of group 11, along with evidence of a mild post-chemotherapy response, that suggesting a partial response to treatment (**Figure 2F**). Notably, groups 7 and 12 exhibited extensive necrosis, stromal fibrosis with cholesterol crystals, and hemosiderin deposition, suggestive of prior tumor involvement. However, no residual tumor cells were detected, indicating effective tumor clearance following conversion therapy (**Supplementary Figure 1**). Final pathology confirmed ypT0N1M0 IIA and achieved MPR, which met the Expert Consensus of Chinese Medical Association of containing less than 10% viable tumor cells in excised tissue following conversion therapy (10). The patient recovered uneventfully and received four cycles of adjuvant chemoimmunotherapy (pembrolizumab 200 mg, cisplatin 75 mg/m² + nab-paclitaxel 260 mg/m², q3w) starting June 12, 2021, followed by 2-year pembrolizumab maintenance. Serial peripheral blood circulating tumor DNA (ctDNA) monitoring detected no molecular residual disease (**Figure 4**). Regular imaging surveillance (chest/abdominal CT, brain MRI, PET-CT) showed no recurrence, with ongoing PFS exceeding 50 months. The gene list of NGS tissue and ctDNA is provided in the **Supplementary Table 1**.

## Discussion

For driver-negative stage IV squamous NSCLC, immune checkpoint inhibitor (ICI) plus chemotherapy has become the first-line standard treatment regimen (11). KEYNOTE-407 five-follow-up data demonstrated pembrolizumab + chemotherapy improved median PFS (8.0 vs. 5.1 months) and OS (17.2 vs. 11.6 months) in SqCC, regardless of PD-L1 status (3). However, evidence regarding immunotherapy efficacy in squamous NSCLC with brain metastases remains scarce, as most trials excluded these patients or enrolled only those with stable metastases. The blood-brain barrier limits intracranial ICI efficacy, with historical single-agent ICI objective response rates (ORR) of 16%-33% (12). Pooled analysis of KEYNOTE-021, -189, and -407 revealed superior median OS (18.8 vs. 7.6 months) and PFS (6.9 vs. 4.1 months) for pembrolizumab-chemotherapy versus chemotherapy in NSCLC patients with brain metastases (13). CheckMate 227 reported that dual immunotherapy (nivolumab + ipilimumab) significantly improved 5-year intracranial PFS rates (16% vs. 6%) (14). Chemotherapy may potentiate ICIs by enhancing T-cell responses and disrupting tumor-associated macrophage (TAM) activity (15), potentially overcoming blood-brain barrier limitations (16).

This case achieved intracranial pCR after three cycles of pembrolizumab + chemotherapy, suggesting intracranial penetration of combined therapy. Such outcomes are exceptionally rare in brain metastatic squamous NSCLC, where reported intracranial pCR rates with chemoimmunotherapy remain below 5% (13, 17). The patient’s high PD-L1 expression (TPS=75%) and TMB-H (28.49 Mut/Mb) likely contributed to this response, as both biomarkers correlate positively with immunotherapy efficacy. KEYNOTE-042 established median OS of 20.0 months for PD-L1 TPS ≥50% versus 12.2 months with chemotherapy (18), while TMB-H (≥10 Mut/Mb) associates with ≥40% ICI response rates (19, 20). Synergistic effects of PD-L1 and TMB may amplify antitumor immunity by enhancing T-cell infiltration and activity (21), potentially breaching the blood-brain barrier. Notably, spatial and temporal heterogeneity exists between primary NSCLC and brain metastases in tumor immune microenvironments (22), with intensified immunosuppression in intracranial lesions (23). Bischof et al. demonstrated significantly higher extracranial versus intracranial PD-L1 TPS (p=0.013) in matched samples, with longer intracranial PFS (54.8 vs. 15.4 months) in patients with high intracranial PD-L1 (≥40%) (24). In this case, the brain lesions reached pCR, and the status of PD-L1 and TMB of the brain lesions could not be further confirmed.

The role of surgery in systemically treated stage IV squamous NSCLC remains debated. Dong Tian’s team advocates conversion surgery as a promising strategy to improve outcomes (25). The research of Haibin Wang shows that the median OS of patients with stage IVA who received surgical treatment reached 28.6 months, which was significantly better than that of the non-surgical group (16.2 months) (26). Successful surgical conversion in this case aligns with emerging evidence that PD-L1-high/TMB-H status predicts favorable responses to immunochemotherapy. However, the feasibility of surgery after conversion therapy depends on the biological characteristics of the tumor. Currently, only a few cases have shown that patients with stage IV lung squamous cell carcinoma can be downstaged to an operable condition through the combination of immunotherapy and chemotherapy. Moreover, high PD-L1 expression or high tumor mutational burden (TMB-H) may be the key predictive factors for surgery after successful conversion (27). In this case, the pulmonary lesion significantly regressed after the combination of immunotherapy and chemotherapy (from 8.9 cm to 3.6 cm). The postoperative pathology confirmed MPR, but residual lesions still remained in the interlobar lymph nodes (group 11). Based on this, 4 cycles of chemotherapy combined with immunotherapy were carried out after surgery, followed by maintenance with single-agent immunotherapy for up to 2 years. This phenomenon suggests that surgery can resect radiologically visible drug-resistant clones, while systemic treatment (such as postoperative adjuvant immunotherapy) can further control micrometastases. The success of this case indicates that for specific populations (oligometastasis, high immune activity markers), the “sandwich” model (drug + surgery + drug) may become a strategy to break through the survival bottleneck. Anyway, this still requires verification through prospective studies.

Brain metastases tend to recur relatively easily after surgery. Therefore, the standard treatment for resectable brain metastases is postoperative SRS after surgical resection. But this patient remains recurrence-free for >50 months without SRS, possibly attributable to immunotherapy’s “tail effect”—sustained immune-mediated eradication of residual cells. The ctDNA was undetectable prior to surgery after conversion therapy, and the molecular residual disease (MRD) status remained negative throughout the subsequent long-term follow-up. This phenomenon further validates that the ctDNA status can serve as a predictor of the patient’s prognosis. Numerous studies have indicated that, in contrast to imaging examinations, ctDNA can more sensitively predict the recurrence risk of early-stage lung cancer (28). This implies that ctDNA holds a distinctive and crucial significance in assessing the patient’s condition and prognosticating the outcome.

There are also some issues during the conversion therapy. After the conversion therapy with the combination of immunotherapy and chemotherapy, PET-CT indicates that the tumor is active with increased radioactive uptake. However, after resection, no cancer residue is found. This inconsistency in results interferes with clinical diagnosis and also highlights the limitations of imaging examinations in determining the true state of the tumor. Existing research has confirmed (29) that the clearance of ctDNA is more accurate than imaging examinations in predicting pCR, and patients with persistent negative postoperative MRD often have a longer survival period. Therefore, for patients with brain lesions achieving pCR after immunotherapy and ctDNA clearance, whether SRS treatment can be waived becomes a question worthy of exploration. Due to the existence of the blood-brain barrier, ctDNA in the blood may not comprehensively and accurately reflect the true state of brain lesions (30). Therefore, in the future, attempting to combine the detection of blood ctDNA and cerebrospinal fluid ctDNA may provide a more reliable basis for the decision-making of postoperative SRS treatment. This direction awaits further exploration.

In addition, the withdrawal time of single-agent immunotherapy after surgery for patients with brain metastases is also worthy of further discussion. Long-term immunotherapy may bring certain adverse reactions and economic burdens. How to determine the optimal withdrawal time while ensuring the treatment effect is an urgent problem to be solved in clinical practice. Formulating a more precise and individualized treatment plan based on MRD test results is expected to provide better medical services for patients. However, the realization of this goal still requires a large amount of research and rich clinical data as support.

In conclusion, we report the first case of a lung squamous cell carcinoma patient with brain metastases, high PD-L1 expression and high TMB, who achieved a PFS of over 50 months after surgery following the combination of immunotherapy and chemotherapy. This case suggests that precision medicine guided by biomarkers such as PD-L1, TMB, and MRD, combined with a multidisciplinary comprehensive strategy, may bring the possibility of radical treatment for patients with advanced NSCLC. In the future, it is necessary to further optimize the treatment mode and withdrawal criteria to achieve the best balance between efficacy and safety.

## Data Availability

The original contributions presented in the study are included in the article/**Supplementary Material**. Further inquiries can be directed to the corresponding author.
