# Correction: A stage IV lung squamous cell cancer patient with brain metastases, high PD-L1 &TMB, achieves pCR and long-term survival after immune-chemotherapy and radical surgery: a case report and literature review

**DOI:** 10.3389/fimmu.2025.1660020

**Published:** 2025-07-22

**Authors:** Xiangyu Xu, Zheng Ma

**Affiliations:** Department of Thoracic Surgery, Chongqing General Hospital, Chongqing University, Chongqing, China

**Keywords:** non-small cell lung cancer (NSCLC), brain metastasis, programmed cell death ligand 1, tumor mutational burden (TMB), pathological complete response (PCR), circulating tumor DNA (ctDNA)

In the published article, there was an error in affiliation 1. Instead of “Department of Thoracic Surgery, Chongqing People’s Hospital, Chongqing, China”, it should be “Department of Thoracic Surgery, Chongqing General Hospital, Chongqing University, Chongqing, China”.

The authors apologize for this error and state that this does not change the scientific conclusions of the article in any way. The original article has been updated.

