# Supplementary material for: A stage IV lung squamous cell cancer patient with brain metastases, high PD-L1 & TMB, achieves pCR and long-term survival after immune-chemotherapy and radical surgery: a case report and literature review
Source: Front Immunol. 2025 Jul 4;16:1601125. doi: 10.3389/fimmu.2025.1601125 (PMC12271089; doi:10.3389/fimmu.2025.1601125)
Supplement: Supplementary file 1 [file DataSheet1.pdf]

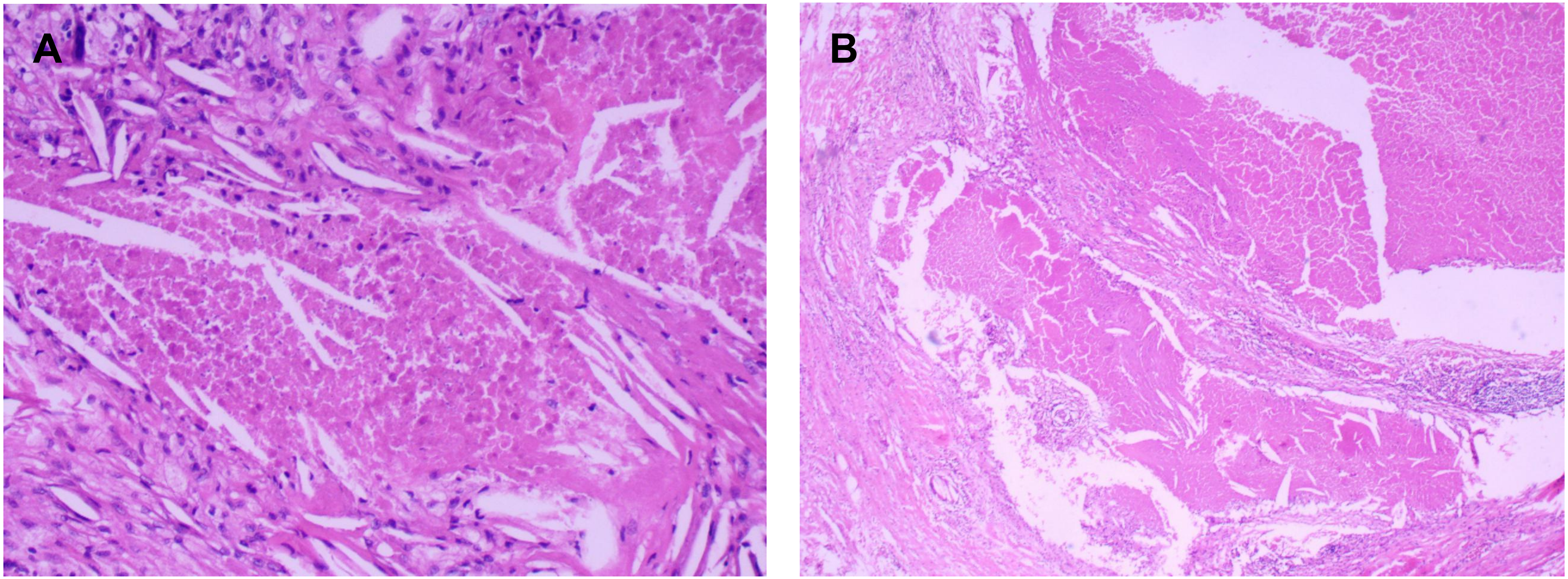

Supplementary Figure 1: Pathological results of lymph nodes: A. group 7 lymph nodes, the maximum diameter of the original metastasis was 13mm, no residual cancer was found, the area of the original metastasis was necrotic, and the interstitial fibrous tissue was proliferated with cholesterol crystals and iron yellow deposition, which was consistent with the response after treatment; B. group 12 lymph nodes, the maximum diameter of the original metastatic lesions was 8mm, with no residual cancer, large area necrosis with inflammatory cell infiltration, interstitial fibrous tissue proliferation with cholesterol crystals and iron yellow deposition, which was consistent with the response after treatment.
